# Neuronal reprogramming improves brain health in TgAD and aging rats

**DOI:** 10.1002/alz70855_107476

**Published:** 2025-12-25

**Authors:** Jessica Ribeiro, Mary Hill, Tina Beckett, Joanne Mclaurin

**Affiliations:** ^1^ Sunnybrook Research Institute, Toronto, ON, Canada; ^2^ University of Toronto, Toronto, ON, Canada

## Abstract

**Background:**

Cognitive decline, however, is not driven by disturbances in a few synapses in isolation, but by an aberration in the functioning of the entire neuronal network, which is assembled through interactions between excitatory, inhibitory and neuromodulatory cells. As disease progresses, loss of specific populations of neurons exacerbates cognitive function and renders a point of no return for present therapeutic interventions. Furthermore the neuroinflammatory response within the brain contributes to ongoing neuronal damage, and thus strategies to limit toxic inflammatory responses should benefit also slow disease progression. Our overall goal is to utilize neuronal reprogramming of toxic astrocytes into neurons as a means of decreasing neuroinflammation, stabilizing neuronal network function and ultimately cognition.

**Method:**

To elicit neuronal reprograming, proneural transcription factors (TFs) ASCL‐1 or mutant ASCL‐1 SA6 were expressed in 14‐ and 18‐month‐old TgF344 AD and their non‐transgenic littermate rats using an AAV2/5 vector under a GFAP promoter. TFs were unilaterally injected into the hippocampal hilus and rats were monitored for behavior at seven weeks post TF delivery before pathological examination. Pathological and cognitive function was examined 7 weeks post viral injection.

**Result:**

We demonstrate a significant improvement in cognitive function 7 weeks after neuronal reprogramming, with a subsequent improvement in astrogliosis, microgliosis, neuronal density with a concomitant decrease in amyloid plaque load.

**Conclusion:**

Neuronal reprogramming decreases neuroinflammation and increases survival of neurons within the hippocampus of both TgF344 AD and aging F344 non‐transgenic littermates. We demonstrate the return to a more homeostatic brain environment post‐neuronal reprogramming in middle‐aged and older rats.

